# Supplementary material for: Analysis of PPARγ Signaling Activity in Psoriasis
Source: Int J Mol Sci. 2021 Aug 10;22(16):8603. doi: 10.3390/ijms22168603 (PMC8395241; doi:10.3390/ijms22168603)
Supplement: Supplementary file 1 [file ijms-22-08603-s001.zip › Supplemental materials_Analysis of PPARg signaling activity in psoriasis/Pathway models/Models images and html files/Interleukin-17 and interleukin-22 signaling in psoriasis/9330.html]

MAPK9


# Protein MAPK9

|  |  |
| --- | --- |
| URN | urn:agi-llid:5601 |
| Total Entities | 0 |
| Connectivity | 2770 |
| Name | MAPK9 |
| Description | mitogen-activated protein kinase 9 |
| Notes | The protein encoded by this gene is a member of the MAP kinase family. MAP kinases act as an integration point for multiple biochemical signals, and are involved in a wide variety of cellular processes such as proliferation, differentiation, transcription regulation and development. This kinase targets specific transcription factors, and thus mediates immediate-early gene expression in response to various cell stimuli. It is most closely related to MAPK8, both of which are involved in UV radiation induced apoptosis, thought to be related to the cytochrome c-mediated cell death pathway. This gene and MAPK8 are also known as c-Jun N-terminal kinases. This kinase blocks the ubiquitination of tumor suppressor p53, and thus it increases the stability of p53 in nonstressed cells. Studies of this gene's mouse counterpart suggest a key role in T-cell differentiation. Several alternatively spliced transcript variants encoding distinct isoforms have been reported. [provided by RefSeq, Sep 2008] |
| Primary Cell Localization | Cytoplasm |
| Class | Protein kinase |

---

|  |  |
| --- | --- |
| Pathway | Osteoarthritis |
|  | Skin Aging |
|  | MTHFR Activity Regulation |
|  | Dopamine Receptors Signaling |
|  | T-Cell Dependent B-Cell Activation |
|  | T-Cell Independent B-Cell Activation |
|  | Th1-Cell Differentiation |
|  | Treg-Cell Differentiation |
|  | Th2-Cell Differentiation |
|  | T-Cell Receptor Signaling |
|  | Nociception Expression Targets Signaling |
|  | CNR1/2 -> IL1B/2/4/6/10 Production |
|  | DRD2 Expression Targets |
|  | Proteins Involved in Atherosclerosis |
|  | Dendritic Cells Function in Atherosclerosis |
|  | Th1-Cells Activation and Proliferation in Atherosclerosis |
|  | Myocardial Remodeling |
|  | Myocardial Infarction |
|  | Inflammatory Reaction in Acne Vulgaris |
|  | Atopic Dermatitis Onset |
|  | Mast-Cells Activation in Atopic Dermatitis |
|  | IL17 Signaling in Psoriasis |
|  | IL22 Induces Keratinocyte Proliferation in Psoriasis |
|  | Proteins with Altered Expression in Psoriasis |
|  | B-Cell Activation in Systemic Lupus Erythematosus |
|  | Th2-Cell Function in Systemic Lupus Erythematosus |
|  | Acute Cytotoxic CD8+T-Cell Response against Melanocytes |
|  | T-Cell Cytotoxic Response against Melanocytes in Vitiligo |
|  | Humoral Immunity in Vitiligo |
|  | Treg-Cell Function in Diabetes Mellitus Type 1 |
|  | Natural Killer T-Cell Roles in Diabetes Mellitus Type 1 |
|  | non-Suppressive Treg-Cell in Diabetes Mellitus Type 1 |
|  | TLR2 Signaling in Treg-Cell in Type 1 Diabetes (Animal Model) |
|  | Treg-Cell Activation in Diabetes Mellitus |
|  | Proteins Involved in Diabetes Mellitus Type 2 |
|  | Podocyte Dysfunction in Diabetic Nephropathy |
|  | Proteins Involved in Diabetic Nephropathy |
|  | Proteins Involved in Insulin Resistance |
|  | Insulin Resistance in Myocytes Induced by Obesity |
|  | GNRH Signaling Impairment in Hypogonadotrophic Hypogonadism |
|  | CD4+T-Cell Response in Celiac Disease |
|  | OipA Signaling in Helicobacter Pylori Infection |
|  | Th2-Cell Function in Ulcerative Colitis |
|  | T-Cell Cytotoxic Response in Ulcerative Colitis |
|  | Osteoclast Activation in Multiple Myeloma |
|  | Hepatocellular Carcinoma |
|  | Growth Factor Signaling in Hepatocellular Carcinoma |
|  | Pancreatic Ductal Carcinoma |
|  | Growth Factor Signaling in Pancreatic Neoplasms |
|  | Pancreatic Neuroendocrine Tumors |
|  | TGFBR Signaling in Pancreatic Neoplasms |
|  | Th2-Cell Response in Asthma |
|  | TNF and IL1B Induce Metalloproteinase Synthesis in Osteoarthritis |
|  | Synovial Fibroblast Activation in Psoriatic Arthritis |
|  | Keratinocyte Activation in Psoriatic Arthritis |
|  | PTPN22 Role in Rheumatoid Arthritis |
|  | Synovial Fibroblast Activation by Citokines in Rheumatoid Arthritis |
|  | B-Cells Function in Systemic Scleroderma |
|  | Th2-Cells Function in Systemic Scleroderma |
|  | Skin Fibrosis |
|  | CCR5 Signaling in Macrophages |
|  | CD4+ T-Cell Function Decline in HIV |
|  | CD4+ T-Cell Death in HIV |
|  | Glioma Invasion Signaling |
|  | Proteins Involved in Breast Cancer Related to WNT Signaling Pathway |
|  | Proteins Involved in Breast Cancer Related to ESR1 Signaling Pathway |
|  | WNT Signaling in Breast Cancer |
|  | ESR1/ERBB Positive Luminal Breast Cancer |
|  | ESR1 Signaling in Breast Cancer |
|  | Endometrial Cancer |
|  | Endometrioid Endometrial Cancer |
|  | Clear Cell Endometrial Cancer and Papillary Serous Endometrial Cancer |
|  | Angiogenesis in Endometriosis |
|  | Endometrioid Ovarian Carcinoma |
|  | Clear Cell Ovarian Carcinoma |
|  | High-Grade Serous Ovarian Carcinoma |
|  | Low-Grade Serous Ovarian Carcinoma |
|  | Ovarian Cancer |
|  | Mucinous Ovarian Carcinoma |
|  | Subepithelial Fibroblasts in Middle Ear Cholesteatoma |
|  | HPV E5 Regulates EDNRA and EGFR Signaling Pathway in Keratinocytes |
|  | Androgen Receptor/Akt Signaling |
|  | Steroids Induced Cataract |
|  | WNT Signaling in Cystic Kidney Disease |
|  | Podocyte Dysfunction in Glomerulonephritis |
|  | Apoptosis of Renal Tubule Epithelial Cell in Pyelonephritis |
|  | CD38/CD19 -> JUN/FOS/NF-kB Signaling in B-cell Proliferation |
|  | ANGPT2 -> AP-1/CREBBP/MYC Expression Targets |
|  | AREG -> AP-1 Expression Targets |
|  | Dopamine/Gi Expression Targets |
|  | BTC -> AP-1/ATF/CREB Expression Targets |
|  | EGF -> AP-1/ATF Expression Targets |
|  | EGF -> CREB/CREBBP/ELK/SRF/MYC Expression Targets |
|  | EGF -> TP53 Expression Targets |
|  | EGFR/ERBB2 -> TP53 Signaling |
|  | EGFR -> AP-1/ATF2 Signaling |
|  | EGFR -> AP-1/CREB/ELK/SRF/MYC Signaling |
|  | EREG -> AP-1/ATF Expression Targets |
|  | Erythropoietin -> AP-1/MYC/CREB Expression Targets |
|  | F2 -> AP-1/CREB/ELK/SRF/SP1 Expression Targets |
|  | PLG -> AP-1/CREB/ELK/SRF/SP1 Expression Targets |
|  | FGF9 -> AP-1/CREB/MYC Expression Targets |
|  | FGF10 -> AP-1/CREB/CREBBP/MYC Expression Targets |
|  | FGF1 -> AP-1/CREB/ELK/SRF/MYC Expression Targets |
|  | FGF8 -> AP-1/CREB/MYC Expression Targets |
|  | FGF18 -> AP-1/CREB Expression Targets |
|  | FGF4 -> AP-1/MYC Expression Targets |
|  | FGF7 -> AP-1/CREB/CREBBP/MYC Expression Targets |
|  | FGF2 -> AP-1/CREB/CREBBP/ELK/SRF/MYC Expression Targets |
|  | FGFR -> AP-1/CREB/CREBBP/ELK/SRF/MYC Signaling |
|  | FrizzledR -> JUN/PAX2 Signaling |
|  | HBEGF -> AP-1/ATF Expression Targets |
|  | HBEGF -> TP53 Expression Targets |
|  | HBEGF -> CREB/MYC Expression Targets |
|  | HGF -> AP-1/CREB/ELK/SRF/MYC Expression Targets |
|  | HGFR -> AP-1/CREB/MYC Signaling |
|  | RAS/RAF/MAPK Signaling Activation in Cancer |
|  | RAS/RAF/MAPK Signaling Activation by Blocking of Tumor Suppressors |
|  | Apoptosis Block by Death Receptor Signaling in Cancer |
|  | Apoptosis Evasion in Cancer: Overview |
|  | Apoptosis Block by BCL2 Family Proteins in Cancer |
|  | VEGFA Dependent Angiogenesis in Cancer |
|  | VEGF Independent Angiogenesis in Cancer |
|  | FibronectinR -> AP-1/ELK/SRF/SREBF Signaling |
|  | CTGF -> AP-1/CREB/MYC Expression Targets |
|  | PAF/Gq -> AP-1/ATF1/CREB/ERK/SRF Expression Targets |
|  | Dronabinol/Anandamide Expression Targets |
|  | S1P Expression Targets |
|  | NRG1 -> CREB/CREBBP/ELK/SRF/MYC Expression Targets |
|  | NRG1 -> TP53 Expression Target |
|  | NRG1 -> AP-1/ATF Expression Targets |
|  | NGF -> AP-1/TP53/MYC Expression Targets |
|  | BDNF Expression Targets |
|  | NTF3 Expression Targets |
|  | NTF4 Expression Targets |
|  | PDGFB -> AP-1/CREB/MYC Expression Target |
|  | PDGFD -> AP-1 Expression Targets |
|  | PDGF -> AP-1/CREB/CREBBP/MYC Expression Targets |
|  | PDGFR -> AP-1/MYC Signaling |
|  | AVP/Gs -> CREB/ELK/SRF/AP-1/EGR Expression Targets |
|  | GNRH1 Expression Targets |
|  | GNRH2 Expression Targets |
|  | EDN1 Expression Targets |
|  | EDN3 Expression Targets |
|  | AVP/Gq -> CREB/ELK/SRF/AP-1/EGR Expression Targets |
|  | AngiopoietinR -> AP-1 Signaling |
|  | NTRK -> AP-1/CREB/ELK/SRF/MYC/SMAD3/TP53 Signaling |
|  | GAS6 -> AP-1/CREB Expression Targets |
|  | FIGF -> AP-1 Expression Target |
|  | KITLG -> AP-1/CREB/CREBBP/MYC Expression Targets |
|  | CSF1 -> AP-1/CREB/CREBBP/MYC Expression Targets |
|  | PGF -> AP-1/CREB/CREBBP/MYC Expression Targets |
|  | FLT3LG -> AP-1/CREB/CREBBP Expression Targets |
|  | TGFBR -> AP-1 Signaling |
|  | TNF-alpha/TNFRSF1A Signaling |
|  | JNK/MAPK Signaling |
|  | VEGF Signaling |
|  | EGFR Signaling |
|  | WNT Planar Cell Polarity (PCP) non-Canonical Signaling |
|  | T-Cell Receptor -> AP-1 Signaling |
|  | TGFA -> TP53 Expression Targets |
|  | TGFA/AP-1/ATF Expression Targets |
|  | TGFA -> CREB/CREBBP/ELK-SRF/MYC Expression Targets |
|  | TGFB3-TGFBR2 Expression Targets |
|  | TGFB2-TGFBR2 Expression Targets |
|  | TGFB1-TGFBR2 Expression Targets |
|  | FASLG Expression Targets |
|  | TNFSF10 Expression Targets |
|  | LTA Expression Targets |
|  | TNFSF14 Expression Targets |
|  | TNF -> AP-1 Expression Targets |
|  | CD72 -> AP-1 Expression Targets |
|  | CD40LG -> NF-kB/ELK/SRF -> CREB/NFATC Expression Targets |
|  | TNFSF13 Expression Targets |
|  | TNFSF13B Expression Targets |
|  | TNF -> ELK-SRF Expression Targets |
|  | TNF -> TP53/ATF Expression Targets |
|  | EDA Expression Targets |
|  | EctodysplasinR -> AP-1 Signaling |
|  | TNFR -> CREB/ELK-SRF Signaling |
|  | TNFR -> AP-1/ATF/TP53 Signaling |
|  | NGFR -> AP-1/CEBPB/CREB/ELK/SRF/TP53 Signaling |
|  | TNFRSF1A -> AP-1/ATF/TP53 Signaling |
|  | TNFRSF1A -> CREB/ELK-SRF Signaling |
|  | Thrombopoietin -> AP-1/CREB/CREBBP/MYC Expression Targets |
|  | TLR4 -> AP-1 Expression Targets |
|  | TLR5 Expression Targets |
|  | TLR7 Expression Targets |
|  | TLR4 -> AP-1/EGR1/HIF1A Expression Targets |
|  | TLR1 -> 2/6 Expression Targets |
|  | TLR9 Expression Targets |
|  | TLR -> AP-1 Signaling |
|  | VEGFA -> AP-1/CREBBP/MYC Expression Targets |
|  | VEGFR -> AP-1/CREB/MYC Signaling |
|  | WNT1 Expression Targets |
|  | WNT3A Expression Targets |
|  | WNT4 Expression Targets |
|  | WNT5A Expression Targets |
|  | WNT7A Expression Targets |
|  | 1\_Inflammation in keratinocytes in the pathology of acne |
|  | 2\_Acute phase initiation in atopic dermatitis |
|  | 2\_Interleukin-17 and interleukin-22 signaling in psoriasis |
|  | 1\_The onset of atopic dermatitis |
|  | 1\_TNF and IL1B provoke ECM degradation in osteoarthritis |
|  | 1\_Osteoclast-mediated bone resorption in postmenopausal women |
|  | 1\_Formation of cysts in polycystic kidney disease |
|  | 2\_Podocyte dysfunction in glomerulonephritis |
|  | 1\_Disruption of luteinizing hormone and follicle-stimulating hormone secretion in PCOS |
|  | 2\_Intestine epithelial cells drive immunological responses in ulcerative colitis |
|  | 4\_2\_Insulin resistance: inflammation-related insulin resistance |
|  | 4\_3\_Insulin resistance: FFA-related insulin resistance |
|  | 2\_Neuron and Schwann cell death |
|  | Interleukin-17 and interleukin-22 signaling in psoriasis |

---

|  |  |
| --- | --- |
| Group | Genes with Mutations Associated with Ageing (non-Mammalian Animal Model) |

---

|  |  |
| --- | --- |
| Source | curated |
|  | GO component |

---

|  |  |
| --- | --- |
| MedScan ID | 5601 |

---

|  |  |
| --- | --- |
| LocusLink ID | 5601 |
|  | 26420 |
|  | 50658 |
|  | 50657 |
|  | 19100 |
|  | 103866 |
|  | 534125 |
|  | 562552 |

---

|  |  |
| --- | --- |
| Alias | mitogen activated protein kinase 9 |
|  | stress-activated protein kinase 1a |
|  | MK09\_HUMAN |
|  | protein kinase mitogen-activated 9 |
|  | c-Jun N-terminal kinase 2 |
|  | SAPK-alpha |
|  | MK09 |
|  | mitogen-activated protein kinase 9 isoform JNK2 alpha2 |
|  | OTTMUSP00000005726 |
|  | p54-alpha |
|  | JNK/SAPK alpha |
|  | stress activated protein kinase alpha II |
|  | Stress-activated protein kinase JNK2 |
|  | OTTMUSP00000005727 |
|  | c-Jun kinase 2 |
|  | SAPK1a |
|  | OTTMUSP00000005725 |
|  | RP23-10G21.6 |
|  | JNK-55 |
|  | PRKM9 |
|  | OTTMUSP00000005728 |
|  | OTTHUMP00000161543 |
|  | c-Jun N-terminal kinase II |
|  | p54JNK2 |
|  | MAP kinase 9 |
|  | JNK2BETA |
|  | c-Jun kinase II |
|  | OTTHUMP00000161542 |
|  | Jun kinase 2 |
|  | JNK2ALPHA |
|  | JNK/SAPK |
|  | AI851083 |
|  | p54aSAPK |
|  | Jun kinase II |
|  | JNK2 |
|  | kinase JNK2 |
|  | MAPK9 |
|  | JNK2A |
|  | JNK2B |
|  | SAPK |
|  | p54a |
|  | Jun kinase |
|  | MAPK 9 |
|  | mitogen-activated protein kinase 9 |
|  | protein kinase, mitogen-activated 9 |
|  | Stress-activated protein kinase JNK2 |
|  | protein kinase mitogen-activated 9 (MAP kinase) |
|  | IL23R |
|  | interleukin-23 receptor |
|  | MAPK9. |
|  | protein kinase JNK2 |

---

|  |  |
| --- | --- |
| EC Number | 2.7.11.24 |
|  | 2.7.1.- |
|  | 2.7.1.37 |

---

|  |  |
| --- | --- |
| GO ID | 0005524 |
|  | 0004705 |
|  | 0004707 |
|  | 0004712 |
|  | 0008134 |
|  | 0038095 |
|  | 0007254 |
|  | 0007258 |
|  | 0071276 |
|  | 0071310 |
|  | 0034614 |
|  | 0035556 |
|  | 0048666 |
|  | 0018105 |
|  | 0043065 |
|  | 2001235 |
|  | 0010628 |
|  | 0010744 |
|  | 0071803 |
|  | 0031398 |
|  | 1901485 |
|  | 0061833 |
|  | 0006468 |
|  | 0051090 |
|  | 0042752 |
|  | 0010468 |
|  | 0009612 |
|  | 0048511 |
|  | 0005737 |
|  | 0005829 |
|  | 0005739 |
|  | 0043005 |
|  | 0005654 |
|  | 0005634 |
|  | 0008656 |
|  | 0016301 |
|  | 0031435 |
|  | 0000166 |
|  | 0004672 |
|  | 0004674 |
|  | 0016740 |
|  | 0034644 |
|  | 0031175 |
|  | 0016310 |
|  | 0010770 |
|  | 0032722 |
|  | 0043280 |
|  | 0045429 |
|  | 0051770 |
|  | 0031394 |
|  | 0032308 |
|  | 0001934 |
|  | 0045893 |
|  | 0046328 |
|  | 0031396 |
|  | 0001836 |
|  | 0046686 |
|  | 0042493 |
|  | 0043204 |
|  | 0000165 |
|  | 0006919 |
|  | 0071474 |
|  | 1904646 |
|  | 0071363 |
|  | 0071347 |
|  | 0071222 |
|  | 0071356 |
|  | 0007417 |
|  | 0006626 |
|  | 0014075 |
|  | 1990089 |
|  | 0010033 |
|  | 0009636 |
|  | 0009414 |
|  | 0006950 |
|  | 0005622 |
|  | 0002755 |
|  | 0002756 |
|  | 0035666 |
|  | 0045087 |
|  | 0051403 |
|  | 0034166 |
|  | 0034134 |
|  | 0034138 |
|  | 0034142 |
|  | 0034146 |
|  | 0034162 |
|  | 0038123 |
|  | 0038124 |
|  | 0002224 |
|  | 0016772 |
|  | 0008063 |
|  | 0034130 |
|  | 0023014 |
|  | 0044445 |
|  | 0031558 |
|  | 0008152 |
|  | 0051773 |
|  | 0051789 |
|  | 0007165 |
|  | 0005515 |
|  | 0045941 |
|  | 0034097 |
|  | 0032496 |
|  | 0004713 |
|  | 0016020 |
|  | 0004682 |
|  | 0004691 |

---

|  |  |
| --- | --- |
| KEGG ID | hsa:5601 |
|  | mmu:26420 |
|  | rno:50658 |

---

|  |  |
| --- | --- |
| MedLine Reference | 10381549 |
|  | 10471813 |
|  | 10490605 |
|  | 10523642 |
|  | 10629060 |
|  | 10669748 |
|  | 10797012 |
|  | 10811224 |
|  | 11024282 |
|  | 11042694 |
|  | 11057897 |
|  | 11062067 |
|  | 11172026 |
|  | 11278395 |
|  | 11278789 |
|  | 11359773 |
|  | 11527157 |
|  | 11566021 |
|  | 11602244 |
|  | 11948398 |
|  | 11959861 |
|  | 12023963 |
|  | 12110590 |
|  | 12167088 |
|  | 12171600 |
|  | 12189133 |
|  | 12225289 |
|  | 12417588 |
|  | 12477932 |
|  | 12538493 |
|  | 12743126 |
|  | 12788955 |
|  | 12818176 |
|  | 14504284 |
|  | 14724588 |
|  | 15158451 |
|  | 15262983 |
|  | 15326485 |
|  | 15474087 |
|  | 15489334 |
|  | 15527495 |
|  | 15637069 |
|  | 15655348 |
|  | 15693750 |
|  | 15778501 |
|  | 15860507 |
|  | 15911620 |
|  | 1602151 |
|  | 16169070 |
|  | 16189514 |
|  | 16381010 |
|  | 16412424 |
|  | 16552729 |
|  | 17074809 |
|  | 17192257 |
|  | 17296730 |
|  | 17397142 |
|  | 602896 |
|  | 7737129 |
|  | 7969172 |
|  | 8001819 |
|  | 8548291 |
|  | 8626452 |
|  | 8649858 |
|  | 8654373 |
|  | 8945519 |
|  | 9130707 |
|  | 9374467 |
|  | 9393873 |
|  | 9405416 |
|  | 9628874 |
|  | 9732264 |
|  | 10021384 |
|  | 10230788 |
|  | 10349636 |
|  | 10498280 |
|  | 10559486 |
|  | 10674476 |
|  | 10922068 |
|  | 11042159 |
|  | 11076861 |
|  | 11157052 |
|  | 11217851 |
|  | 11358804 |
|  | 11423995 |
|  | 11435459 |
|  | 11466342 |
|  | 11562351 |
|  | 11920420 |
|  | 11927625 |
|  | 11927626 |
|  | 12147621 |
|  | 12223406 |
|  | 12376563 |
|  | 12447443 |
|  | 12466851 |
|  | 12486099 |
|  | 12689591 |
|  | 12697041 |
|  | 12756228 |
|  | 12857751 |
|  | 12897243 |
|  | 14517246 |
|  | 14578290 |
|  | 14633627 |
|  | 14657393 |
|  | 14704147 |
|  | 14704277 |
|  | 15013949 |
|  | 15102471 |
|  | 15123655 |
|  | 15272135 |
|  | 15350216 |
|  | 15375216 |
|  | 15528206 |
|  | 15566943 |
|  | 15567863 |
|  | 15572149 |
|  | 15572687 |
|  | 15673436 |
|  | 15767678 |
|  | 15778378 |
|  | 15805466 |
|  | 15816869 |
|  | 15867147 |
|  | 16043490 |
|  | 16081685 |
|  | 16113653 |
|  | 16141072 |
|  | 16141073 |
|  | 16217033 |
|  | 16314504 |
|  | 16466687 |
|  | 16514068 |
|  | 16571730 |
|  | 16579967 |
|  | 16606357 |
|  | 16627484 |
|  | 16818881 |
|  | 16973441 |
|  | 16973625 |
|  | 17056565 |
|  | 17143273 |
|  | 17167049 |
|  | 17202416 |
|  | 17301033 |
|  | 17301952 |
|  | 17337450 |
|  | 17365404 |
|  | 17400176 |
|  | 17449011 |
|  | 17644613 |
|  | 8817558 |
|  | 8889548 |
|  | 9806643 |
|  | 9878062 |
|  | 12445686 |
|  | 12559955 |
|  | 15504737 |
|  | 16641100 |
|  | 17333127 |
|  | 17555943 |
|  | 8177321 |
|  | 9651196 |

---

|  |  |
| --- | --- |
| Organism | Homo sapiens {Organism urn:agi-taxid:9606} |
|  | Mus musculus {Organism urn:agi-taxid:10090} |
|  | Rattus norvegicus {Organism urn:agi-taxid:10116} |
|  | Homo sapiens |
|  | Mus musculus |
|  | Rattus norvegicus |
|  | Bos taurus |
|  | Danio rerio |

---

|  |  |
| --- | --- |
| Mouse chromosome position | 11 |
|  | 11 B1.2 |

---

|  |  |
| --- | --- |
| OMIM ID | 602896 |
|  | OMIM:602896 |

---

|  |  |
| --- | --- |
| Rat chromosome position | 10q21 |
|  | 10q22 |

---

|  |  |
| --- | --- |
| Hugo ID | 6886 |
|  | HGNC:6886 |

---

|  |  |
| --- | --- |
| Human chromosome position | 5q35.3 |
|  | 5q35 |

---

|  |  |
| --- | --- |
| Swiss-Prot Accession | P45984 |
|  | D7R525 |
|  | D7R526 |
|  | P45984.2 |
|  | Q8C094 |
|  | Q9WTU6 |
|  | Q5NCK8 |
|  | Q9WTU6.2 |
|  | D4A5V8 |
|  | P49186 |
|  | Q6P727 |
|  | A0A0G2JYS4 |
|  | P49186.1 |
|  | A8K0S3 |
|  | B5BU66 |
|  | B5M0B4 |
|  | D3DWQ8 |
|  | D3DWQ9 |
|  | Q15708 |
|  | Q15710 |
|  | Q15711 |
|  | Q8N5C5 |
|  | Q5NCK9 |
|  | Q5NCL5 |
|  | Q8C097 |
|  | Q8VDD2 |
|  | Q9WTU4 |
|  | Q9WTU5 |
|  | Q5NCK7 |
|  | Q3V398 |
|  | MK09\_HUMAN |

---

|  |  |
| --- | --- |
| PIR ID | A55480 |
|  | S71102 |
|  | S43968 |

---

|  |  |
| --- | --- |
| GenBank ID | NC\_000005 |
|  | NM\_002752 |
|  | NP\_002743 |
|  | NM\_139070 |
|  | NP\_620709 |
|  | NM\_001364613 |
|  | NP\_001351542 |
|  | NM\_001364608 |
|  | NP\_001351537 |
|  | NM\_001364609 |
|  | NP\_001351538 |
|  | NM\_001364611 |
|  | NP\_001351540 |
|  | NM\_001364612 |
|  | NP\_001351541 |
|  | NM\_139068 |
|  | NP\_620707 |
|  | NM\_139069 |
|  | NP\_620708 |
|  | NM\_001364607 |
|  | NP\_001351536 |
|  | XM\_005265940 |
|  | XP\_005265997 |
|  | XM\_017009642 |
|  | XP\_016865131 |
|  | NM\_001135044 |
|  | NP\_001128516 |
|  | NM\_001364610 |
|  | NP\_001351539 |
|  | XM\_017009643 |
|  | XP\_016865132 |
|  | NM\_001308244 |
|  | NP\_001295173 |
|  | NG\_029059 |
|  | AC008610 |
|  | AC104115 |
|  | CH471165 |
|  | EAW53757 |
|  | EAW53758 |
|  | EAW53759 |
|  | EAW53760 |
|  | EAW53761 |
|  | EAW53762 |
|  | EAW53763 |
|  | EAW53764 |
|  | EAW53765 |
|  | DQ066599 |
|  | AAY46156 |
|  | AB451302 |
|  | BAG70116 |
|  | AB451355 |
|  | BAG70169 |
|  | AB451433 |
|  | BAG70247 |
|  | AK289638 |
|  | BAF82327 |
|  | BC032539 |
|  | AAH32539 |
|  | BF056535 |
|  | BQ025589 |
|  | CR536580 |
|  | CAG38817 |
|  | CX758487 |
|  | DB451562 |
|  | EU927388 |
|  | ACH57450 |
|  | HM036706 |
|  | ADH10365 |
|  | HM036707 |
|  | ADH10366 |
|  | HM036708 |
|  | ADH10367 |
|  | HM036709 |
|  | ADH10368 |
|  | L31951 |
|  | AAA56831 |
|  | U09759 |
|  | AAA74740 |
|  | U34821 |
|  | AAC50606 |
|  | U35002 |
|  | AAC50608 |
|  | U35003 |
|  | AAC50609 |
|  | P45984 |
|  | NC\_000077 |
|  | NM\_016961 |
|  | NP\_058657 |
|  | NM\_001163671 |
|  | NP\_001157143 |
|  | NM\_001163672 |
|  | NP\_001157144 |
|  | NM\_207692 |
|  | NP\_997575 |
|  | XM\_017314563 |
|  | XP\_017170052 |
|  | XM\_017314562 |
|  | XP\_017170051 |
|  | AJ315339 |
|  | CAC88132 |
|  | AJ315340 |
|  | AJ315341 |
|  | AJ315342 |
|  | AJ315343 |
|  | AJ315344 |
|  | AJ315345 |
|  | AJ315346 |
|  | AJ315347 |
|  | AJ315348 |
|  | AJ315349 |
|  | AJ315350 |
|  | AL606479 |
|  | CH466575 |
|  | EDL33733 |
|  | EDL33734 |
|  | EDL33735 |
|  | AB005664 |
|  | BAA85876 |
|  | AF052466 |
|  | AAD22576 |
|  | AF052467 |
|  | AAD22577 |
|  | AF052468 |
|  | AAD22578 |
|  | AF052469 |
|  | AAD22579 |
|  | AK031959 |
|  | BAC27623 |
|  | AK031974 |
|  | BAC27634 |
|  | AK043169 |
|  | BAE43319 |
|  | AK090057 |
|  | AK145083 |
|  | BAE26227 |
|  | BB362351 |
|  | BC024514 |
|  | BC028341 |
|  | AAH28341 |
|  | CX235052 |
|  | Q9WTU6 |
|  | NC\_005109 |
|  | NM\_001270544 |
|  | NP\_001257473 |
|  | NM\_001270545 |
|  | NP\_001257474 |
|  | NM\_017322 |
|  | NP\_059018 |
|  | XM\_017597479 |
|  | XP\_017452968 |
|  | XM\_006246318 |
|  | XP\_006246380 |
|  | AC\_000078 |
|  | AABR07029576 |
|  | AAHX01063750 |
|  | CH473948 |
|  | EDM04237 |
|  | EDM04238 |
|  | EDM04239 |
|  | EDM04240 |
|  | EDM04241 |
|  | EDM04242 |
|  | BC061870 |
|  | AAH61870 |
|  | BI282175 |
|  | DN948189 |
|  | FQ234999 |
|  | L27111 |
|  | AAA42108 |
|  | L27112 |
|  | AAA42109 |
|  | P49186 |
|  | XM\_006714891 |
|  | XP\_006714954 |
|  | XM\_011534594 |
|  | XP\_011532896 |
|  | NC\_018916 |
|  | AMYH02012903 |
|  | DQ893713 |
|  | ABM84639 |
|  | EU176194 |
|  | ABW03645 |
|  | XM\_006533379 |
|  | XP\_006533442 |
|  | AC\_000033 |
|  | AAHY01093196 |
|  | AAHY01093197 |
|  | XM\_006246317 |
|  | XP\_006246379 |
|  | AC\_000137 |
|  | ABBA01010059 |
|  | ABBA01010060 |
|  | ABBA01010061 |
|  | ABBA01010062 |
|  | ABBA01010063 |
|  | CAI23940 |
|  | CAI23941 |
|  | CAI23942 |
|  | CAI23943 |
|  | NT\_023133 |
|  | NW\_922818 |
|  | NW\_001838967 |
|  | AC\_000048 |
|  | CR612106 |
|  | CR614221 |
|  | CR619061 |
|  | NT\_096135 |
|  | NW\_001030461 |
|  | Q3V398 |
|  | Q5NCK7 |
|  | Q5NCK8 |
|  | Q8C094 |
|  | NW\_047334 |
|  | NW\_001084656 |
|  | Q6P727 |
|  | Q5NCK9 |
|  | Q5NCL5 |
|  | 119574142 |
|  | 119574143 |
|  | 119574144 |
|  | 119574145 |
|  | 119574146 |
|  | 119574147 |
|  | 119574148 |
|  | 119574149 |
|  | 119574150 |
|  | 123994075 |
|  | 1463129 |
|  | 1463133 |
|  | 1463135 |
|  | 21618470 |
|  | 49168644 |
|  | 598183 |
|  | 607786 |
|  | 66394592 |
|  | 85700366 |
|  | 123792343 |
|  | 13124366 |
|  | 148701786 |
|  | 148701787 |
|  | 148701788 |
|  | 18254507 |
|  | 20306844 |
|  | 26327765 |
|  | 26327787 |
|  | 4558391 |
|  | 4558393 |
|  | 4558395 |
|  | 4558397 |
|  | 56205898 |
|  | 56205899 |
|  | 56205900 |
|  | 56205901 |
|  | 6141553 |
|  | 74203059 |
|  | 74217232 |
|  | 81886186 |
|  | 81886187 |
|  | 81886188 |
|  | 81886191 |
|  | 81913852 |
|  | AW108403 |
|  | BG068409 |
|  | AI851083 |
|  | 1346338 |
|  | 149052420 |
|  | 149052421 |
|  | 149052422 |
|  | 149052423 |
|  | 149052424 |
|  | 149052425 |
|  | 38197448 |
|  | 493207 |
|  | 493209 |
|  | 81885302 |
|  | Q8N5C5 |
|  | NW\_000039 |
|  | NT\_086684 |
|  | 122135977 |
|  | 86438360 |
|  | AAI12748 |
|  | BC112747 |
|  | NC\_007305 |
|  | NM\_001046369 |
|  | NP\_001039834 |
|  | NW\_001495309 |
|  | Q2KI72 |
|  | NC\_007134 |
|  | NW\_001512526 |
|  | XM\_685934 |
|  | XP\_691026 |
|  | AABR06063744 |

---

|  |  |
| --- | --- |
| Swiss-Prot ID | MK09\_HUMAN |
|  | MK09\_MOUSE |
|  | MK09\_RAT |
|  | Q5NCK8\_MOUSE |
|  | Q8C094\_MOUSE |
|  | B5M0B4\_HUMAN |
|  | Q5NCK7\_MOUSE |
|  | A8K0S3\_HUMAN |
|  | B5BU66\_HUMAN |
|  | Q5NCK9\_MOUSE |
|  | D3DWQ8\_HUMAN |
|  | D3DWQ9\_HUMAN |

---

|  |  |
| --- | --- |
| Cell Localization | Cytoplasm |
|  | Nucleus |
|  | Plasma membrane |

---

|  |  |
| --- | --- |
| Ensembl ID | ENSG00000050748 |
|  | ENSP00000397422.2 |
|  | ENST00000425491.6 |
|  | ENSP00000443149.1 |
|  | ENST00000539014.5 |
|  | ENSP00000394560.2 |
|  | ENST00000452135.6 |
|  | ENSP00000377028.3 |
|  | ENST00000393360.7 |
|  | ENSP00000345524.6 |
|  | ENST00000343111.10 |
|  | ENSP00000389338.1 |
|  | ENST00000455781.5 |
|  | ENSMUSG00000020366 |
|  | ENSMUSP00000136977.1 |
|  | ENSMUST00000178543.7 |
|  | ENSMUSP00000132864.1 |
|  | ENSMUST00000164643.7 |
|  | ENSMUSP00000104808.2 |
|  | ENSMUST00000109179.8 |
|  | ENSMUSP00000020634.7 |
|  | ENSMUST00000020634.13 |
|  | ENSRNOG00000002823 |
|  | ENSRNOP00000004010.3 |
|  | ENSRNOT00000004010.4 |
|  | ENSRNOP00000003987.3 |
|  | ENSRNOT00000003987.5 |
|  | ENSRNOP00000070771.1 |
|  | ENSRNOT00000083688.1 |
|  | ENSP00000397422 |
|  | ENST00000425491 |
|  | ENSP00000394560 |
|  | ENST00000452135 |
|  | ENSP00000377028 |
|  | ENST00000393360 |
|  | ENSP00000345524 |
|  | ENST00000343111 |
|  | ENSP00000389338 |
|  | ENST00000455781 |
|  | ENSP00000443149 |
|  | ENST00000539014 |
|  | ENSMUSP00000136977 |
|  | ENSMUST00000178543 |
|  | ENSMUSP00000132864 |
|  | ENSMUST00000164643 |
|  | ENSMUSP00000104808 |
|  | ENSMUST00000109179 |
|  | ENSMUSP00000020634 |
|  | ENSMUST00000020634 |
|  | ENSRNOP00000004010 |
|  | ENSRNOT00000004010 |
|  | ENSRNOP00000003987 |
|  | ENSRNOT00000003987 |
|  | ENSRNOP00000070771 |
|  | ENSRNOT00000083688 |

---

|  |  |
| --- | --- |
| MGI ID | MGI:1346862 |
|  | 1346862 |
|  | 1202396 |
|  | 2144306 |

---

|  |  |
| --- | --- |
| RGD ID | 628847 |

---

|  |  |
| --- | --- |
| Unigene ID | Hs.484371 |
|  | Mm.68933 |
|  | Rn.9910 |
|  | Rn.177202 |
|  | Hs.654461 |
|  | Hs.348446 |
|  | Bt.52287 |
|  | Dr.34373 |

---

|  |  |
| --- | --- |
| Homologene ID | 55685 |

---

|  |  |
| --- | --- |
| Shape | Sickle-vertex |

---

|  |  |
| --- | --- |
| IPI ID | IPI00191806 |
|  | IPI00231634 |
|  | IPI00024673 |
|  | IPI00220382 |
|  | IPI00220383 |
|  | IPI00303550 |
|  | IPI00123875 |
|  | IPI00223339 |
|  | IPI00223340 |
|  | IPI00223342 |
|  | IPI00913982 |
|  | IPI00653963 |
|  | IPI00777753 |

---

|  |  |
| --- | --- |
| Microarray ID | 101362\_at |
|  | 203218\_at |
|  | 1421877\_at |

---

|  |  |
| --- | --- |
| Homo sapiens Chromosome position | 5q35 |

---

|  |  |
| --- | --- |
| Ensembl Transcript ID | ENST00000393362 |
|  | ENST00000347470 |
|  | ENST00000343111 |
|  | ENST00000393360 |

---

|  |  |
| --- | --- |
| ENSEMBL | ENSG00000050748 |

---

|  |  |
| --- | --- |
| FunctionalClass | ATP binding |
|  | JUN kinase |
|  | MAP kinase |
|  | nucleotide binding |
|  | protein binding |
|  | protein serine/threonine kinase |
|  | transferase |
|  | kinase |
|  | protein kinase |
|  | protein-tyrosine kinase |
|  | cAMP-dependent protein kinase |
|  | protein kinase CK2 |

---

|  |  |
| --- | --- |
| Bos taurus Chromosome position | 7 |

---

|  |  |
| --- | --- |
| Mus musculus Chromosome position | 11 B1.2 |
|  | 11 |
|  | 11 B1.3 |

---

|  |  |
| --- | --- |
| KEGG pathway | Adipocytokine signaling pathway |
|  | Colorectal cancer |
|  | ErbB signaling pathway |
|  | Fc epsilon RI signaling pathway |
|  | Focal adhesion |
|  | GnRH signaling pathway |
|  | Insulin signaling pathway |
|  | MAPK signaling pathway |
|  | Pancreatic cancer |
|  | Toll-like receptor signaling pathway |
|  | Type II diabetes mellitus |
|  | Wnt signaling pathway |

---

|  |  |
| --- | --- |
| Rattus norvegicus Chromosome position | 10q22 |

---

|  |  |
| --- | --- |
| Ensembl Gene ID | ENSG00000050748 |

---

|  |  |
| --- | --- |
| ProteinAtlas NormalTissue | Tissue: adrenal gland; Cell type: glandular cells; Level: Strong; Expression type: Staining; Reliability: Uncertain |
|  | Tissue: appendix; Cell type: glandular cells; Level: Moderate; Expression type: Staining; Reliability: Uncertain |
|  | Tissue: appendix; Cell type: lymphoid tissue; Level: Moderate; Expression type: Staining; Reliability: Uncertain |
|  | Tissue: bone marrow; Cell type: hematopoietic cells; Level: Negative; Expression type: Staining; Reliability: Uncertain |
|  | Tissue: breast; Cell type: glandular cells; Level: Strong; Expression type: Staining; Reliability: Uncertain |
|  | Tissue: bronchus; Cell type: respiratory epithelial cells; Level: Strong; Expression type: Staining; Reliability: Uncertain |
|  | Tissue: cerebellum; Cell type: cells in granular layer; Level: Moderate; Expression type: Staining; Reliability: Uncertain |
|  | Tissue: cerebellum; Cell type: cells in molecular layer; Level: Strong; Expression type: Staining; Reliability: Uncertain |
|  | Tissue: cerebellum; Cell type: Purkinje cells; Level: Strong; Expression type: Staining; Reliability: Uncertain |
|  | Tissue: cerebral cortex; Cell type: glial cells; Level: Negative; Expression type: Staining; Reliability: Uncertain |
|  | Tissue: cerebral cortex; Cell type: neuronal cells; Level: Strong; Expression type: Staining; Reliability: Uncertain |
|  | Tissue: cervix, uterine; Cell type: glandular cells; Level: Negative; Expression type: Staining; Reliability: Uncertain |
|  | Tissue: cervix, uterine; Cell type: squamous epithelial cells; Level: Negative; Expression type: Staining; Reliability: Uncertain |
|  | Tissue: colon; Cell type: glandular cells; Level: Strong; Expression type: Staining; Reliability: Uncertain |
|  | Tissue: duodenum; Cell type: glandular cells; Level: Strong; Expression type: Staining; Reliability: Uncertain |
|  | Tissue: epididymis; Cell type: glandular cells; Level: Strong; Expression type: Staining; Reliability: Uncertain |
|  | Tissue: esophagus; Cell type: squamous epithelial cells; Level: Weak; Expression type: Staining; Reliability: Uncertain |
|  | Tissue: fallopian tube; Cell type: glandular cells; Level: Moderate; Expression type: Staining; Reliability: Uncertain |
|  | Tissue: gall bladder; Cell type: glandular cells; Level: Strong; Expression type: Staining; Reliability: Uncertain |
|  | Tissue: heart muscle; Cell type: myocytes; Level: Negative; Expression type: Staining; Reliability: Uncertain |
|  | Tissue: hippocampus; Cell type: glial cells; Level: Negative; Expression type: Staining; Reliability: Uncertain |
|  | Tissue: hippocampus; Cell type: neuronal cells; Level: Strong; Expression type: Staining; Reliability: Uncertain |
|  | Tissue: kidney; Cell type: cells in glomeruli; Level: Strong; Expression type: Staining; Reliability: Uncertain |
|  | Tissue: kidney; Cell type: cells in tubules; Level: Moderate; Expression type: Staining; Reliability: Uncertain |
|  | Tissue: lateral ventricle; Cell type: glial cells; Level: Negative; Expression type: Staining; Reliability: Uncertain |
|  | Tissue: lateral ventricle; Cell type: neuronal cells; Level: Negative; Expression type: Staining; Reliability: Uncertain |
|  | Tissue: liver; Cell type: bile duct cells; Level: Weak; Expression type: Staining; Reliability: Uncertain |
|  | Tissue: liver; Cell type: hepatocytes; Level: Negative; Expression type: Staining; Reliability: Uncertain |
|  | Tissue: lung; Cell type: macrophages; Level: Moderate; Expression type: Staining; Reliability: Uncertain |
|  | Tissue: lung; Cell type: pneumocytes; Level: Moderate; Expression type: Staining; Reliability: Uncertain |
|  | Tissue: lymph node; Cell type: germinal center cells; Level: Strong; Expression type: Staining; Reliability: Uncertain |
|  | Tissue: lymph node; Cell type: non-germinal center cells; Level: Strong; Expression type: Staining; Reliability: Uncertain |
|  | Tissue: nasopharynx; Cell type: respiratory epithelial cells; Level: Strong; Expression type: Staining; Reliability: Uncertain |
|  | Tissue: oral mucosa; Cell type: squamous epithelial cells; Level: Moderate; Expression type: Staining; Reliability: Uncertain |
|  | Tissue: ovary; Cell type: follicle cells; Level: Strong; Expression type: Staining; Reliability: Uncertain |
|  | Tissue: ovary; Cell type: ovarian stroma cells; Level: Weak; Expression type: Staining; Reliability: Uncertain |
|  | Tissue: pancreas; Cell type: exocrine glandular cells; Level: Strong; Expression type: Staining; Reliability: Uncertain |
|  | Tissue: pancreas; Cell type: islets of Langerhans; Level: Weak; Expression type: Staining; Reliability: Uncertain |
|  | Tissue: placenta; Cell type: decidual cells; Level: Moderate; Expression type: Staining; Reliability: Uncertain |
|  | Tissue: placenta; Cell type: trophoblastic cells; Level: Strong; Expression type: Staining; Reliability: Uncertain |
|  | Tissue: prostate; Cell type: glandular cells; Level: Moderate; Expression type: Staining; Reliability: Uncertain |
|  | Tissue: rectum; Cell type: glandular cells; Level: Strong; Expression type: Staining; Reliability: Uncertain |
|  | Tissue: salivary gland; Cell type: glandular cells; Level: Moderate; Expression type: Staining; Reliability: Uncertain |
|  | Tissue: skeletal muscle; Cell type: myocytes; Level: Weak; Expression type: Staining; Reliability: Uncertain |
|  | Tissue: skin; Cell type: epidermal cells; Level: Negative; Expression type: Staining; Reliability: Uncertain |
|  | Tissue: small intestine; Cell type: glandular cells; Level: Strong; Expression type: Staining; Reliability: Uncertain |
|  | Tissue: smooth muscle; Cell type: smooth muscle cells; Level: Weak; Expression type: Staining; Reliability: Uncertain |
|  | Tissue: spleen; Cell type: cells in red pulp; Level: Moderate; Expression type: Staining; Reliability: Uncertain |
|  | Tissue: spleen; Cell type: cells in white pulp; Level: Moderate; Expression type: Staining; Reliability: Uncertain |
|  | Tissue: stomach, lower; Cell type: glandular cells; Level: Moderate; Expression type: Staining; Reliability: Uncertain |
|  | Tissue: stomach, upper; Cell type: glandular cells; Level: Strong; Expression type: Staining; Reliability: Uncertain |
|  | Tissue: testis; Cell type: cells in seminiferus ducts; Level: Strong; Expression type: Staining; Reliability: Uncertain |
|  | Tissue: testis; Cell type: Leydig cells; Level: Weak; Expression type: Staining; Reliability: Uncertain |
|  | Tissue: thyroid gland; Cell type: glandular cells; Level: Strong; Expression type: Staining; Reliability: Uncertain |
|  | Tissue: tonsil; Cell type: germinal center cells; Level: Moderate; Expression type: Staining; Reliability: Uncertain |
|  | Tissue: tonsil; Cell type: non-germinal center cells; Level: Strong; Expression type: Staining; Reliability: Uncertain |
|  | Tissue: tonsil; Cell type: squamous epithelial cells; Level: Strong; Expression type: Staining; Reliability: Uncertain |
|  | Tissue: urinary bladder; Cell type: urothelial cells; Level: Moderate; Expression type: Staining; Reliability: Uncertain |
|  | Tissue: uterus, post-menopause; Cell type: cells in endometrial stroma; Level: Negative; Expression type: Staining; Reliability: Uncertain |
|  | Tissue: uterus, post-menopause; Cell type: glandular cells; Level: Strong; Expression type: Staining; Reliability: Uncertain |
|  | Tissue: uterus, pre-menopause; Cell type: cells in endometrial stroma; Level: Negative; Expression type: Staining; Reliability: Uncertain |
|  | Tissue: uterus, pre-menopause; Cell type: glandular cells; Level: Moderate; Expression type: Staining; Reliability: Uncertain |
|  | Tissue: vagina; Cell type: squamous epithelial cells; Level: Negative; Expression type: Staining; Reliability: Uncertain |
|  | Tissue: vulva/anal skin; Cell type: epidermal cells; Level: Negative; Expression type: Staining; Reliability: Uncertain |
|  | Tissue: uterus |
|  | post-menopause; Cell type: cells in endometrial stroma; Level: Negative; Expression type: Staining; Reliability: Uncertain |
|  | Tissue: seminal vesicle; Cell type: glandular cells; Level: Moderate; Expression type: Staining; Reliability: Uncertain |
|  | pre-menopause; Cell type: glandular cells; Level: Moderate; Expression type: Staining; Reliability: Uncertain |

---
